# Fungal Diseases Caused by Serious Contamination of Pharmaceuticals and Medical Devices, and Rapid Fungal Detection Using Nano-Diagnostic Tools: A Critical Review

**DOI:** 10.1007/s00284-023-03506-7

**Published:** 2023-11-17

**Authors:** Mohamed Abd El-Gawad El-Sayed Ahmed, Heba S. Abbas, Muddukrishnaiah Kotakonda

**Affiliations:** 1 Department of Microbiology and Immunology, Faculty of Pharmaceutical Science and Drug Manufacturing, Misr University for Science and Technology, Cairo, 6th of October City, Egypt; 2 Microbiology Department, Egyptian Drug Authority, Previously National Organization for Drug Control and Research, Giza, Egypt; 3 Department of Pharmaceutics, Jamia Salafiya Pharmacy College, Pulikkal, Malappuram District, Kerala India

## Abstract

Fungal-contaminated compounded pharmaceuticals and medical devices pose a public health problem. This review aimed to provide an organized overview of the literature on that critical issue. Firstly, it was found that compounding pharmacies can produce drugs that are contaminated with fungi, leading to outbreaks of severe fungal diseases. Secondly, inadequate sterile compounding techniques or storage conditions, or exceeding the limit of a fungal count, can result in fungal contamination. Lastly, nanotools can be used to rapidly detect fungi, thus improving fungal diagnostic procedures. To achieve this goal, we have reviewed the published data on PubMed, the CDC, and FDA Web sites, and a literature search was undertaken to identify severe fungal infections associated with compounding pharmacies outside of hospitals, limited by the dates 2003 to 2021. The “Preferred Reporting Items for Critical Reviews” were followed in searching, including, and excluding papers. Fungal outbreaks have been documented due to contaminated pharmaceuticals and medical devices. In 2013, 55 people died from fungal meningitis caused by contaminated steroid injections containing methylprednisolone acetate. Additionally, in 2021, *Aspergillus penicillioides* contamination was reported in ChloraPrep drugs, which was attributed to the storage conditions that were conducive to the growth of this fungus. These incidents have resulted in severe infectious diseases, such as invasive mycoses, cornea infections, Endophthalmitis, and intestinal and gastric mycosis. By implementing preventive measures and policies, it is possible to avoid these outbreaks. Creating Nano-diagnostics presents a major challenge, where promptly diagnosing fungal infections is required to determine the proper corrective and preventive measures.

## Introduction

Pharmacies along the manufacturing and distribution pathways of medications are responsible for compounding, repackaging, and labeling sterile medications such as injectables and eye drops. Under the supervision of licensed pharmacist, licensed physicians or other personnel in an outsourcing facility mix, combine, or alter pharmaceutical ingredients to create medications tailored to the needs of individual patients, as mandated by the Food and Drug Administration (FDA) [1]. The advancement of pharmacy compounding practices has created new risks to patient safety. However, it has long been established that pharmacy safety and quality standards guarantee the secure preparation of sterile medications [2]. Nevertheless, some compounding pharmacies need to adhere to these standards, and they did not initially intend to address compounding activities on this scale. In turn, compounding pharmacies have increasingly been implicated in significant healthcare outbreaks caused by contaminated products [2, 3].

Pharmaceutical products can be classified into two microbiologically groups: sterile and non-sterile. The pharmacopoeial monographs specify what microbiological standards must be met for sterile and non-sterile drugs [4]. Specifically, pharmacopeial studies ensure a drug is safe and therapeutically effective [5].

As a group of microorganisms, fungi play a significant role in medicine. Furthermore, numerous infections are caused by fungi, especially in immunocompromised individuals. These organisms constitute a significant threat to human health, as well as contaminating surfaces and spoiling pharmaceuticals, cosmetics, and food products [6].

Pharma manufacturers can suffer serious losses due to fungus contamination of their products, and customers can also suffer serious health problems. In the past, mold and yeast have been reported in numerous pharmaceutical cleanrooms, cold rooms, and controlled areas. As a result of rising ambient temperatures and problems with items brought into cleanrooms, some vaccine and pharmaceutical companies in Europe have increased mold contamination. Moreover, several authors have reported fungi contaminating pharmaceuticals, and recall data collected by the FDA from more than 100 products for 2000–2010, indicating that fungi existed in 21% of products [7].

The most common causes of fungal meningitis include the hematogenous spread (from a heart valve or pulmonary focus), direct extension from the sinuses, or contaminated injection through trauma or the spinal cord [8, 9]. However, Shah et al., 2018 hypothesized that the patient injected fungus while using illicit drugs, which then seeded the meninges, a strategy described earlier for *Cryptococcal meningitis* caused by *Cryptococcus* and cerebral mucormycosis by *Rhizopus arrhizus* [10–12]. Typically, more than fifty per cent of patients who develop fungal meningitis die [9]. A 52% fatality rate for *Aspergillus meningitis* was documented, partly attributable to a high proportion of immunosuppressed individuals with disseminated fungal infection [13].

## Major Fungal Contamination of Pharmaceutical Drugs and Medical Devices

**Fig. 1:**
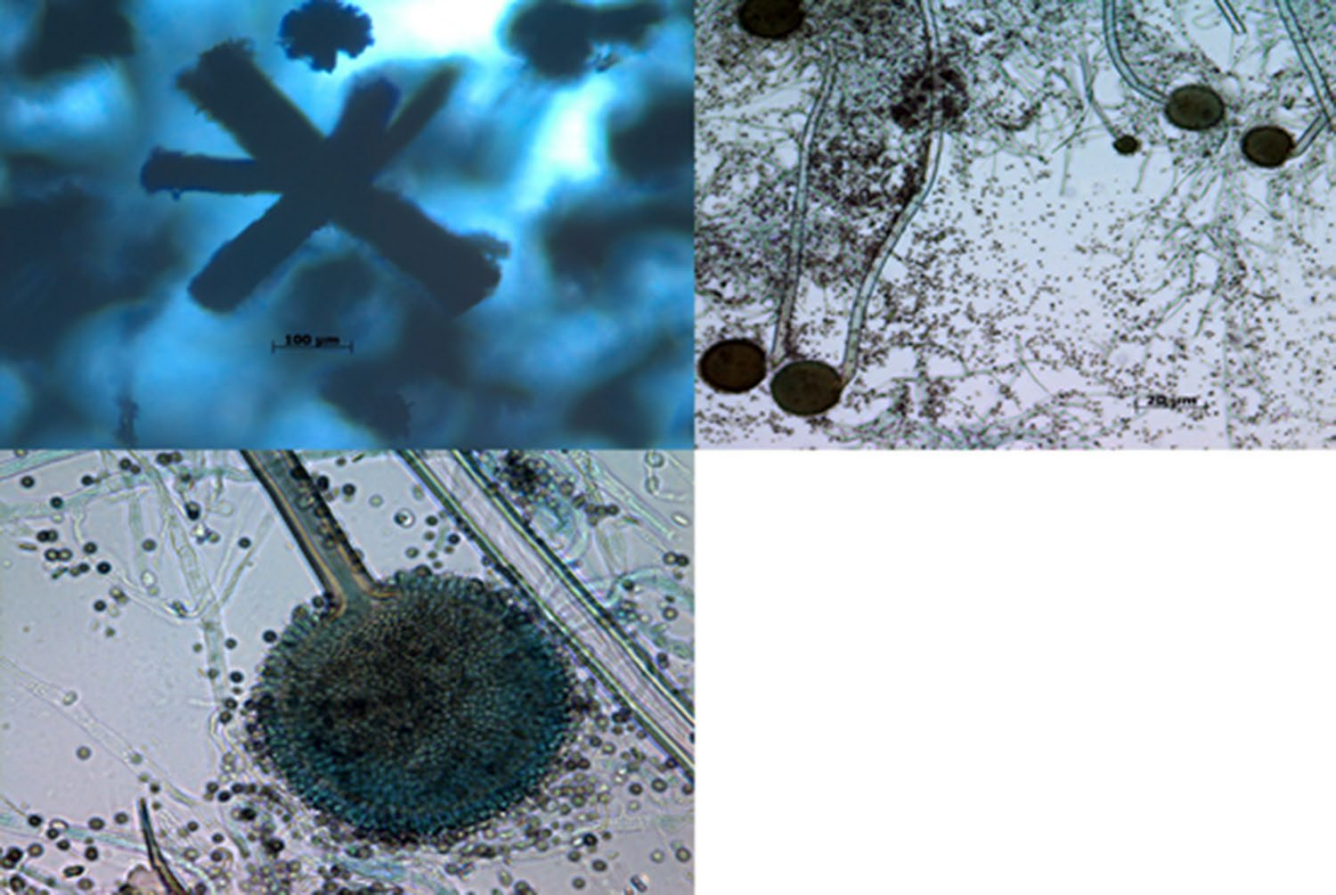
Microscopic examination of *Aspergillus sp.* displayed long chains conidiospores and a chain of conidia is arranged, with the youngest at the bottom and the oldest at the top. (Photos were taken by Muddukrishnaiah K.)

**Fig. 2:**
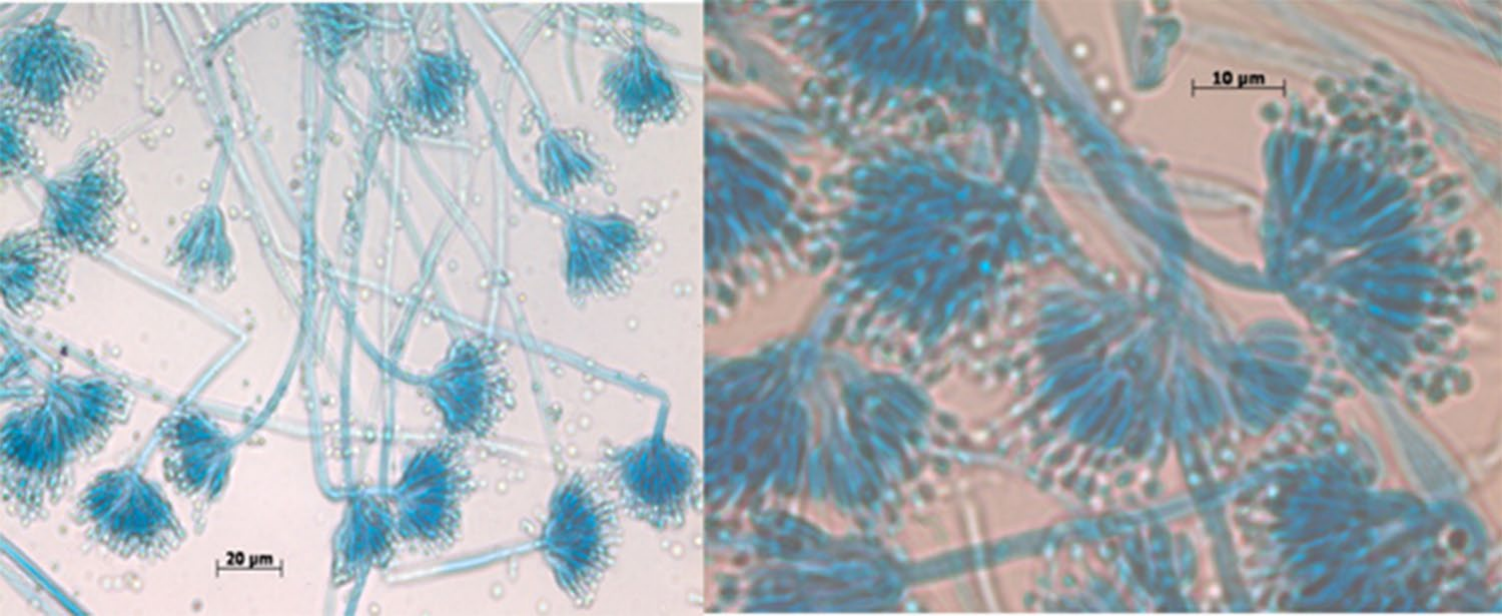
Microscopic examination of *Penicillium sp. Penicillium* conidia on conidiophores. The conidia in long chains of globose shapes, ellipsoids, cylinders or fusiform shapes, and they are arranged in dry chains, divergent or arranged in columns. (Photos were taken by Muddukrishnaiah K.)

**Fig. 3:**
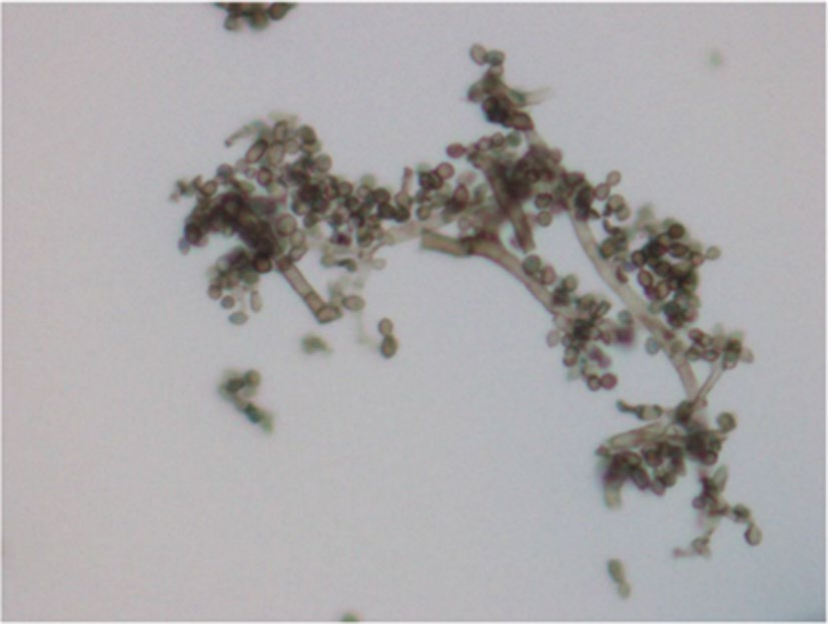
*Exophiala spp.* microscopical characteristics. Septate hyphae with several annellides and conidiogenous loci bearing elliptical conidia of approximately 2–3 mm or 4–5 mm in diameter. (Photo was taken from Marques et al., 2022)

In 2012, FDA and CDC reported fungal contamination in unopened vials of betamethasone and triamcinolone solutions distributed and recalled from the New England Compound Center (NECC). These include *Aspergillus sp., Cladosporium*, *Alternaria*, *Exophiala*, *and Penicillium* species (Table 1, Figs. 1, 2, 3) [14, 15]. Also, in 2013, NECC, Boston, MA, USA, manufactured steroid injections containing methylprednisolone acetate, and 55 patients died from fungal meningitis. In this instance, the drug was administered via the spinal cord to treat arthritis, leading to receiving contaminated products [16]. Also, in the last three years, FDA recalled several drugs due to fungal contamination. For instance, on April 2021 and August 2020, FDA showed *Aspergillus penicillioides* contamination in ChloraPrep drugs, which Becton, Dickinson and Company manufacture. The manufacturer confirmed that the contamination of ChloraPrepTM 3 mL with *A. penicillioides* occurs if it is stored in regions that are extremely hot and humid for more than six months, where it may be consistently exposed to temperatures of 30°C and 75% relative moisture [17, 18].

**Table 1:** Outbreak of certain pharmacy compounding and drug recalls because of the severity of fungal contamination

Medication	Lot number	Fungal contaminations	References
Betamethasone 6 mg/mL injectable–5 mL per vial	08152012@84	*Penicillium *sp., *Cladosporium sp*.	[14]
Triamcinolone 40 mg/mL injectable–2 mL per vial	08172012@60	*Aspergillus tubingensis*, *Penicillium* *sp*.	[14]
Triamcinolone 40mg/mL injectable–10 mL per vial	08242012@2	*Aspergillus fumigatus*	[14]
Methylprednisolone		*Exophiala*	[21]
Intravenous bags of metronidazole, ondansetron and ciprofloxacin		*Cladosporium*	[22, 23]
Triamcinolone acetonide–Ophthalmic injections		*Bipolaris hawaiiensis*	[24–27]
Methylprednisolone acetate injections	05212012@68; 06292012@26; and 08102012@51	*Exserohilum rostratum*	[28]
Linezolid Injections (600mg /300mL)	NDC 55150–242-51 batch CLZ160007	*Mold contamination*	[29]
Risperdal Consta (Risperidone)		*Alternaria alternata*	[30]
Gel-E Donut pillows and Squishon 2 cushions—devices are used in Neonatal and Pediatric ICU settings		*Cladosporium*	[31]
Ruzurgi®(amifampridine) 10 mg Tablets	NDC: 49938–110-01 Control Number 18038 18030 18079	yeast, mold	[20]
ChloraPrep® 3ml	catalog No 260400 260415 930400 930415	*Aspergillus penicillioides*	[17, 18]
PASTA DE LASSAR ANDROMACO®	17LP117 15PL041 15PL040 15PL039	yeast, mold	[19]

Additionally, on April 2020, FDA published that Pasta De Lassar Andromaco Skin Protectant (MarcasUSA, LLC) is contaminated with high levels of fungi and bacteria. Positive results were associated with a specific lot never sold in the US. Nevertheless, four of the remaining lots are being recalled out of caution due to the amount and type of contamination [19]. Furthermore, on September 2021, FDA notified the over limit of yeast, mold, and aerobic bacteria count in the three lots of Ruzurgi® (amifampridine) 10 mg tablets manufactured by Jacobus Pharmaceutical Company Inc, Plainsboro, New Jersey. It is a concern of severe infections to use the contaminated product in patients with Lambert Eaton Syndrome (LEMS) who are already immunosuppressive cases [20].

## Major Fungal Diseases Caused by Fungal Contamination in Pharmaceuticals

### Fungal Meningitis Caused by *Exserohilum rostratum* Contamination

*Exserohilum rostratum*, a main pathogen, was isolated from patient samples and sealed vials of methylprednisolone acetate. Seven hundred forty-nine cases of *E. rostratum* outbreak-related illness, including 6 fatalities, were reported to the Centers for Disease Control and Prevention between Sept 18, 2012, and Jul 1, 2013 [28]. The outbreak appears to result from direct inoculation of *E. rostratum*-contaminated methylprednisolone into living tissue, and the cause appears to be a gross failure of Good Manufacturing Practice (GMP). This is not the first fungal meningitis caused by contaminated steroid formulations. Several occurrences of fungal meningitis caused by *E. dermatitidis* in individuals receiving injectable steroids emerged ten years ago [32]. Only 36% of 328 cases from the six most affected American states investigated could have a valid laboratory diagnosis made using culture, a molecular analysis, or histology. 81% of individuals had a CNS infection, predominantly meningitis, but others had arachnoiditis. There is little knowledge about treating such infections, primarily when the disease comprises the nervous system [16, 32].

Regardless of the earlier fungal outbreaks, it is obvious that the *E. rostratum* meningitis epidemic is a novel clinical entity with little or no prior clinical experience. A PubMed search for *'E. rostratum*' yielded only a few cases of human disease, most of which were keratitis and skin infections. *E. rostratum* has the potential to be pathogenic, given its ability to infect humans. The rarity of the disease, on the other hand, suggests a high level of resistance to illness brought on by this fungus. Most patients were diagnosed with meningitis, which suggests that we may be dealing with a novel disease that may be caused by the hitherto unidentified synergistic combination of *E. rostratum* neurotropism [32].

The cell wall structure of *E. rostratum*, melanin makes it a dematiaceous, or black mold, characterized by ellipsoidal conidia and has truncate hilum [33]. There are many places where it can be found in the environment, on plant debris, in soil, and water. Generally, human infections are limited to allergic sinusitis, keratitis, and soft-tissue infections. In immunocompromised individuals, invasive infection develops only in rare circumstances [33].

This organism's conidia contain different morphologic traits that facilitate identification. The organism grows easily on a standard fungal culture medium, but sporulation to detect the conidia necessitates using a plant-based medium. Even though the fungi grow easily in the laboratory, examination of the cultures of cerebrospinal fluid and tissues may give false results, as has been the case with previous infections. For the diagnosis of fungal infection, molecular identification was performed, and PCR testing on cerebrospinal fluid was valuable in the outbreak.

### Cornea Infection by Fusarium Keratitis Contamination

In April 2006, the Centers for Disease Control and Prevention (CDC) started multistate research into Fusarium keratitis, which mostly affects contact lens wearers [34]. This update describes epidemiologic findings in this inquiry, which point to a link between Bausch & Lomb's ReNu with MoistureLoc® contact lens solution and this study.

**Fig. 4:**
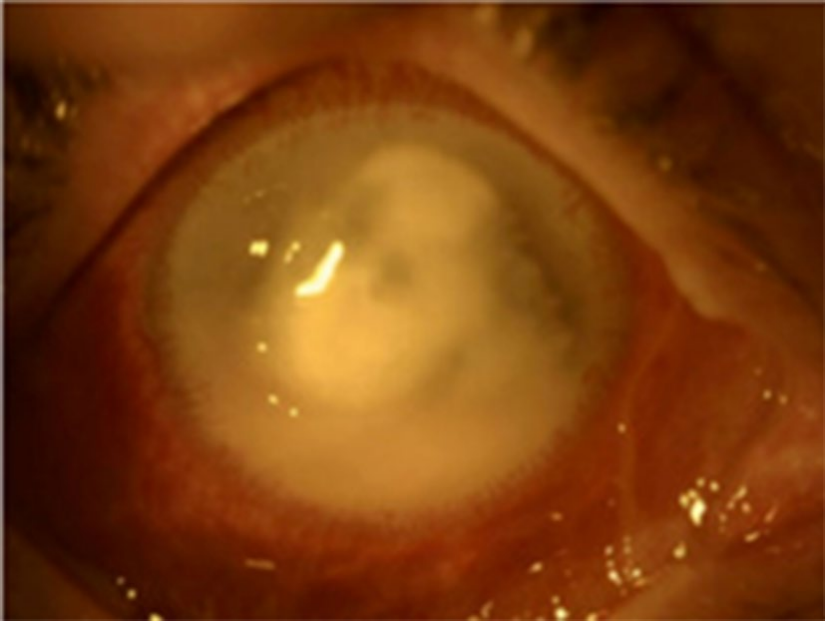
Fungal keratitis caused by *Fusarium spp.* This condition occurs when *Fusarium spp.* infects the cornea (the clear dome covers colored portion of the eye). (Photo was taken from Szaliński et al., 2021)

Fusarium keratitis is a fungal cornea infection generally preceded by eye damage, as shown in Fig. 4 [35]. Although this is not a reportable condition, it is assumed uncommon among contact lens wearers in temperate climates [36]. Fusarium keratitis is treated with antifungal treatment, although it can be critical, resulting in loss of vision and the necessity for corneal transplantation in certain cases [37]. According to the CDC, 130 confirmed cases of Fusarium keratitis infection had been reported since Jun 1, 2005, with no history of recent trauma to the eye and a *Fusarium* species in a corneal biopsy. It was found that among 130 patients with confirmed cases, 125 cases were wearing contact lenses, and 118 cases were able to detect the lens solution they had been using before the beginning of the infection. A global recall and permanent removal of MoistureLoc was declared by Bausch & Lomb (Rochester, New York) on May 15, 2006, following an association between Fusarium keratitis and this contact lens solution [34].

### Intestinal and Gastric Mucormycosis Caused by *Rhizopus microsporus* Contamination

During 6 months in a Queen Mary's hospital, 12 patients on treatment for hematologic malignancies developed an intestinal infection due to *R. microspores*. This fungus was found in several batches of Allopurinol tablets made by the pharmaceutical company, Europharm. Five patients receiving immunosuppressive therapy and being treated for hyperuricemia died of intestinal mucormycosis [38]. Following candidiasis and aspergillosis, zygomycosis is the greatest invasive fungal infection.

Several factors contribute to the rise in zygomycosis, invasion including potent immunosuppression, stem cell and organ transplantation, and possible choice for Zygomycetes after prior treatment with broad-spectrum antifungal drugs, which does not kill them. As part of the manufacturing process, a wet granulation was dried for four hours at 50°C in a tray dryer oven to a water content of 3%. A typical formulation has allopurinol, 100 or 300 mg, corn starch, lactose, magnesium stearate, and povidone (available only in yellow tablets). Corn starch used in tablet manufacture, which contained two CFU of *Rhizopus* per gram, may have been a source of *R. microsporus.* Even though their thermotolerant ascospores and ability to endure for up to four hours at 50°C, and the low water activity, granulations storage at 20°C for five to 14 days before tablet compression and short time sitting indicate that highly contaminated granulations are unlikely [39, 40].

*R. microsporus* has been related to wood tongue depressors [41]. Occurrences of gastric mucormycosis have already been documented in South Africa, presumably as a result of eating food polluted with *Rhizopus*, but the fungus species have not been distinguished [42]. Wooden tongue depressors polluted with *R. microsporus* and utilized to prepare oral drugs induced gastric mycosis outbreak with a 40% mortality [43, 44]. Maraví-Poma et al., 2004 reported that wooden depressors should not be employed in hospitals [43].

### Invasive Mycosis by *Paecilomyces lilacinus* Contaminations

Patients with prolonged neutropenia are at risk for developing invasive mycoses [45]. Usually, the fungal disease is primarily caused by cellular immunity impairment and suppression of the polymorphonuclear leukocyte role [46, 47]. Rarely, *Paecilomyces* species can infect people other than *Aspergillus*. Various *Paecilomyces* are found in soils, decaying vegetation, and animal reservoirs worldwide [48]. During hematology-oncology isolation and bone marrow transplantation at the University Hospital of Basel, Switzerland, Orth et al., 1996 notified an epidemic of *P. lilacinus* infections caused by contaminated skin lotions [49].

In light of these strong epidemiological findings, a second, prolonged screening of closed packages of the skin lotion was undertaken. A total of 12 of 16 sealed bottles were found to contain *P. lilacinus* by the microbiology laboratory. Each lotion milliliter contained 6 and 12,500 colony-forming units (CFU). Preservatives include triclosan (0.3%), chlorhexidine dihydrochloride (0.34%), 36% lipids, and 40 mg of urea per milliliter in the skin lotion. It was discovered that none of the ingredients in the skin lotion was contaminated. In conclusion, *P. lilacinus* was distinguished in the vacant vessels anticipating bottling [49].

The moisturizing lotion is vital for patients with xeroderma and secondary skin toxicity caused by chemotherapy. Using hand lotion regularly after frequent handwashing can prevent dry, irritated skin and dermatitis [50]. Amphotericin B, itraconazole, or fluconazole without antifungal efficacy against *P. lilacinus* [51]. Using skin lotions in hospitals is common, and contaminated products must be detected with a high degree of care.

### Endophthalmitis Caused by *Bipolaris hawaiiensis*-Contaminated Medications

Open globe injury brought on by foreign bodies, lens rupture, or trauma from contaminated items might result in the unusual but potential consequence of post-traumatic endophthalmitis [52]. At least 30 species of fungi belonging to the genus *Bipolaris hawaiiensis*, a dematiaceous mold. There is no doubt that it is a saprophyte and a pathogen of plants [53].

Several cases of intraocular infection by *B. hawaiiensis* from Frankl's Compounding Pharmacy, Ocala, Florida were recently reported by Minckler et al. 2014 [27]. CDC’s Morbidity and Mortality Weekly Report between March and April 2012 describes 33 cases of fungal endophthalmitis in seven states. Additionally, several patients, who received intravitreal combined bevacizumab-triamcinolone injections prepared by the same compounding pharmacy, developed endophthalmitis 41 to 97 days after receiving the injections. Sheyman et al., 2013 highlight the challenges associated with medical diagnosis, identifying the *B. hawaiiensis* as a source of an endophthalmitis outbreak [54]. It is important to note that diabetic macular edema is one of the main causes of vision loss in diabetics. As a means of controlling macular edema and proliferative retinopathy, as well as improving vision, vascular endothelial growth factor inhibitors and corticosteroids (triamcinolone acetonide) are increasingly used intravitreally. It is very common for endophthalmitis to develop after intravitreal injection. It has been reported that between 0.02% and 0.05% of patients with these injections suffer from endophthalmitis [55, 56]. A proper antifungal will not always be able to eradicate these organisms [57]. Thus, the ophthalmologist needs to consider the possibility of fungal etiology when managing a case of endophthalmitis [58]. The global ophthalmic community injects anti-vascular endothelial growth factor and steroids intravitreally many thousands of times per year, especially in diabetic patients, and therefore, compounded drugs should be sterile [27].

## Mycotoxins Associated with Herbal Drugs

In clinical treatment in different parts of the world, herbal medicine has demonstrated a remarkable therapeutic effect, attracting considerable attention from researchers worldwide. The quality and safety of herbal medications have been affected by several cases of fungal and mycotoxin contamination. There have been several instances of mycotoxin and fungus contamination that have impacted the efficacy of herbal drugs. Aflatoxin, ochratoxin A, and fumonisin B are the core mycotoxins present in herbal medications, all of which harm human health [59]. In dried or processed herbal materials, *Penicillium* strains were more prevalent than *Aspergillus* strains. *Aspergillus* strains grow better under high humidity conditions than *Penicillium* strains. While this is happening, herbal medications with high lipid and polysaccharide content may be more prone to contamination from mycotoxins and fungi.

Mycotoxin synthesis can be inhibited successfully using physical, chemical, and microbiological methods. However, some restrictions and issues need to be addressed for physical and chemical prevention. Therefore, a suitable prevention strategy should be suggested based on the characteristics of various herbal medications and different storage circumstances. Recently, COVID-19, a major epidemic disease, have been treated with herbal medications [59, 60]. Therefore, techniques must be created to stop the production of mycotoxins to guarantee the safety of herbal medicines for people.

## Critical Discussion

While performing efficient disinfection and antiseptic operations, only goods that adhere to the verified methodologies described in normative standards should be utilized. The spread of diseases will be stopped if we reach the levels of pathogenic microorganism decrease indicated in the criteria.

It is crucial to identify specific fungal pathogens to create a treatment plan that will be effective and increase the likelihood that the patient will survive. Because different fungal species have varying antifungal susceptibilities, species-level identification is crucial in the management of systemic fungal infections. As a result, a particular antifungal at a certain concentration should be utilized.

There is no FDA-approved protocol for conventional PCR, so results can vary from lab to lab, even though they are rapid and highly sensitive [60–62]. In experienced molecular laboratories where PCR techniques are routinely used, this truth is well known, and the European Aspergillus PCR Initiative endorses standardizing various aspects of testing [63].

To assure a prompt and accurate diagnosis, combinations of the most progressive and advantageous parts of diverse approaches have recently arisen [64]. The main motivation for the birth of these combined techniques, which combine many advantages into one methodology, was the scientific progress that has been felt in recent decades. Examples include PCR combined with electrochemical evaluation and real-time PCR combined with reverse dot blot hybridization. New approaches to diagnosing fungal species, though, are developing, some of which build on the strengths of earlier approaches and others of which are wholly novel.

### Infrared Fourier transform

The fundamentals of spectroscopy provide the foundation of the Fourier transform infrared approach. The functioning of this technology is dependent on exposing the sample to infrared light, part of which is absorbed by the sample. The detector of the apparatus generates a spectrum that serves as the sample’s molecular signature. Clinically speaking, various bacteria will leave behind distinctive fingerprints, and their differentiation is made feasible by analyzing the spectra left behind [65].

### Surface-Enhanced Raman Scattering

Surface-enhanced Raman scattering, which has previously been used to identify a variety of harmful organisms, including fungi, combines Raman spectroscopy and the usage of nanoparticles. This method offers both qualitative and quantitative analysis, enables the tracking of therapeutically significant macromolecules, and creates molecular profiles that can be crucial for assessing the severity of fungus infections [66].

### Solid-Phase Cytometry

Fluorescence microscopy and flow cytometry, two already-used techniques, were combined to create solid-phase cytometry. This ground-breaking technology enables the identification and measurement of numerous microorganisms, including fungi and bacteria. This technology uses clinical samples to immediately offer quick results in an automated manner. However, there are still significant challenges that solid-phase cytometry must overcome in clinical microbiology labs, particularly concerning the validation and verification of the methods [67].

### Nuclear Magnetic Resonance

Using nanoparticles and subsequent magnetic resonance analysis, nuclear magnetic resonance has been effective in the field of microbiology for the identification and detection of species (Peker et al., 2018). In this instance, the target organism is found using beads that have a complementary DNA sequence that allows for binding. With the help of this binding, beads can aggregate and be seen when using magnetic resonance. For product analysis, nuclear magnetic resonance techniques can be utilized independently or after a traditional PCR [68].

### Volatile Organic Compounds Assay

With sensitivity rates above 90%, the volatile organic compounds assay is a novel type of approach for the diagnosis of invasive aspergillosis. In this assay, several *A. fumigatus*-specific metabolites are found in the patient’s exhaled air. This assay is novel in that it makes use of a synthetic olfactory system to distinguish between a variety of volatile organic molecules produced by the pathogen, or “breathprints” [69].

### Biosensors

The use of biosensors constitutes another area of research that is constantly developing. They are intended to be portable devices that transform biological and biochemical data into analytical signals. The careful selection of a specific biomarker of the target pathogen, which must be acceptable for the biological recognition system and has measurably linked traits with normal conditions or with infection, is one of the prerequisites for fungal biosensors manufactured for clinic diagnosis [70].

### Nanotechnology

Nanotechnology has advanced in recent years, allowing the combination of multiple analytical techniques to develop new avenues for research. The electrochemical impedance spectroscopy of membranes allowed the detection of *C. albicans* directly. A detection sensitivity of 10 CFU/mL was demonstrated for anti-Candida antibodies primed on a sensor electrode. To detect *Candida* in clinical samples, the method still requires further modification and optimization. It has been demonstrated that chitosan-stabilized gold nanoparticles can be used as electrochemical biosensors to detect *A. fumigatus.* Toluidine blue was used to generate the electrochemical signal on the sensor probe during several fabrication stages [71]. By using gold nanoparticles modified with a binding peptide recognized through phage display screening, a colourimetric technique was technologically advanced to detect *A. niger* spores. In just 10 min, 50 spores were detected by specific binding peptides [72]. Since fungi rarely produce spores in vivo, this technique is of restricted clinical use, but it could be modified to detect components of hyphal walls. Gold nanoparticle-immobilized protein biomarkers are used in the progress of the next age of biosensors [70, 73].

## Conclusion and Critical View

The contamination of compounded drugs, patient exposure, and public health risks due to breaches in sterile compounding procedures have been highlighted. Additionally, poor handling, repackaging, and nonadherence to good manufacturing practices during dispensing and packaging have resulted in the microbiological contamination of non-sterile pharmaceutical products. This review has provided a brief overview of common fungal contaminants that occur in pharmaceutical cleaning facilities and their corresponding severe human fungal infections. Various genera of fungi have been found to contaminate pharmaceutical products and cause severe diseases. However, due to the difficulty of fungal identification, the examination of fungi by official authorities for microbial quality control is limited. Thus, the microbiologist must rely on a risk assessment based on the kind of product, the cleaning and disinfection practices in place, and the environmental monitoring trend. To prevent such outbreaks, preventive practices and policies must be followed. Nevertheless, detecting fungal contamination in pharmaceutical manufacturing remains a challenge. Nanotechnologies may help to improve current fungal diagnostic practices, as accurate and rapid detection of fungi is often necessary to determine the appropriate corrective actions.

## Data Availability

All data are available with corresponding author.
